# Simple and rapid detection of *Tilletia horrida* causing rice kernel smut in rice seeds

**DOI:** 10.1038/srep33258

**Published:** 2016-09-14

**Authors:** Yu Chen, Xue Yang, Jian Yao, Ei Phyu Kyaw, Ai-Fang Zhang, Yun-Fei Li, Chun-Yan Gu, Hao-Yu Zang, Tong-Chun Gao

**Affiliations:** 1 Institute of Plant Protection and Agro-Products Safety, Anhui Academy of Agricultural Sciences, Hefei 230031, China; 2 Scientific Observing and Experimental Station of Crop Pests in Hefei, Ministry of Agriculture, China; 3 Laboratory of Quality & Safety Risk Assessment for Agro-Products (Hefei), Ministry of Agriculture, China; 4 Anhui Entry-Exit Inspection and Quarantine Bureau, Hefei 230022, China; 5 Department of Biotechnology, Mandalay Technological University, Mandalay, Republic of the Union of Myanmar

## Abstract

A simple and rapid method for the detection of *Tilletia horrida*, the causal agent of rice kernel smut, in rice seeds is developed based on specific polymerase chain reaction (PCR). To design the specific primers for the detection of *T. horrida*, partial sequences of internal transcribed spacer (ITS) DNA region of *T. horrida*, *T. controversa*, *T. walkeri*, *T. ehrhartae*, *T. indica* and *T. caries* were analyzed and compared. A 503-bp fragment was amplified with the designed primers from the *T. horrida* genomic DNA. However, no PCR product was obtained from the DNA of other five *Tilletia* species and 22 fungal plant pathogens tested in the present work indicating the specificity of the primers for the detection of *T. horrida*. The PCR was performed by directly using the spores, isolated from the 21 different rice seed samples, as template DNA. The *T. horrida* was detected in 6 of the samples, indicating that 28.6% of the rice samples were contaminated with the kernel smut pathogen. This simple PCR based diagnostic assay can be applied for the direct and rapid detection and identification of *T. horrida* to screen large numbers of rice seed samples.

The cereal crop rice (*Oryza sativa* L.) is the staple food of over half of the world’s population^1^,^2^ that provides more than 50% of caloric intake^3^. Rice kernel smut (RKS) is caused by fungal pathogen *Tilletia horrida* that was first described in Japan in 1896. It causes a partial bunt that affects both yield and quality of rice by producing black, sooty masses of powdery spores which replace all or part of the grain. The pathogen primarily exists in nature as dark-brown tuberculate teliospores that are widespread in soil overwinter and outside or inside of the host plant seeds. The spores can survive for more than 1 year in soil and for 3 year inside the seed^4^,^5^,^6^. Prior symptom scouting for RKS is not feasible. When symptoms of this disease are identified as present in a farmer’s field, it is too late to apply fungicide for control^7^,^8^.

Previously, the rice kernel disease was considered as a persistent but minor disease^5^,^8^ and remained mostly uninvestigated due to a historical emphasis on major diseases, such as blast and bacterial blight^9^,^10^,^11^. The prevalence of RKS further increased in paddy-irrigated rice areas after the introduction of hybrid rice in the 1970 s. Today, it has worldwide distribution in all the rice-growing countries throughout the Asia, Oceania, Europe, America and Africa^5^,^9^. Every year RKS is accounted for as much as up to a 5% to 20% decrease and 40% to 60% prevalence in diseased grains^6^. Despite the high worldwide rice yield loss caused by *T. horrida*, only limited information is available about its identification, the infection route and chemical control^6^,^12^. Currently, USDA (United States Department of Agriculture) has stipulated that any rice containing more than 3% infected kernels cannot be used as parboiled rice. Producers are docked when infection level in harvested grains is above 3%^7^.

The potential role of China in the global rice system is immense as approximately one-third of all the rice in the world is produced and consumed in China. Rice seeds from China are also being exported to most of the countries in Southeast Asia^13^. However, most of rice cultivars are susceptible to RKS and any possible incursion would cause severer disruption to both the rice production and international trade of China. Furthermore, crop rotation is a common agricultural practice in the rice-growing regions of China. Thus, the presence of related *Tilletia* sp. can cause the ambiguity during leading to raise the trade barriers to rice export.

The current diagnostic protocol for RKS of rice involves the morphological identification of spores, followed by germination to confirm the identity of the pathogen. But these protocols are very time consuming, labor intensive and require highly skilled personnel to distinguish the morphological similar species^14^. Therefore, there is a dire need to develop a simple and rapid method for the detection and identification of *T. horrida* in rice seeds from the other common contaminant fungal species. Traditional PCR based detection methods are simple, rapid, highly specific and sensitive for the target species and can be used to discriminate allelic homologues genomic fragments with minor nucleotide differences. Some of such differences exist in the internal transcribed spacer (ITS) sequences of eukaryotes. ITS region between the nuclear small and large subunit ribosomal DNA has proved useful to distinguish the closely related species of fungi^14^.

This work describes the rapid and accurate PCR based method for the quarantine detection and identification of *T. horrida* in the rice seed samples. The partial ITS region sequences were targeted in the present study to design species specific primers. According to our knowledge this is the first report on the use of conventional PCR based identification and detection of *T. horrida* by using it partial ITS sequences.

## Results

### Primer design and PCR

**Figure 1:**
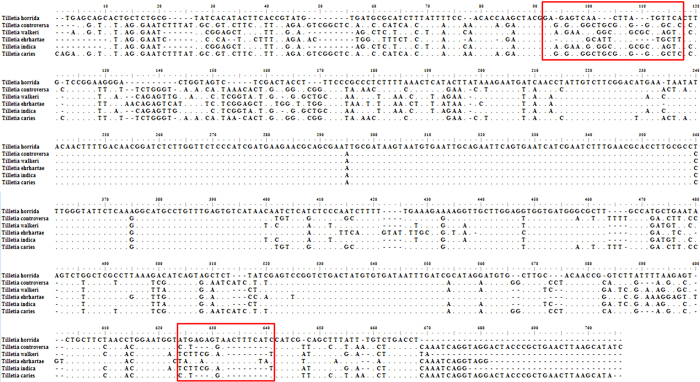
Alignment and analysis of partial sequences of ITS region of 5.8 S rDNA of selected *Tilletia* species. The red frame indicates the nucleotide differences among the six *Tilletia* species.

To design the primer and develop the PCR assay for the detection of *T. horrida,* ITS rDNA sequence of *T. horrida* CN1 (Accession No. DQ827699.1) was used. The sequence was analyzed for homology with the target *Tilletia* species including *T. controversa*, *T. walker*, *T. ehrhartae*, *T. indica* and *T. caries* (Fig. 1). The forward and reverse primers were designed for the amplification of the target region only from *T. horrida*.

### Specificity of the designed primers

**Figure 2:**
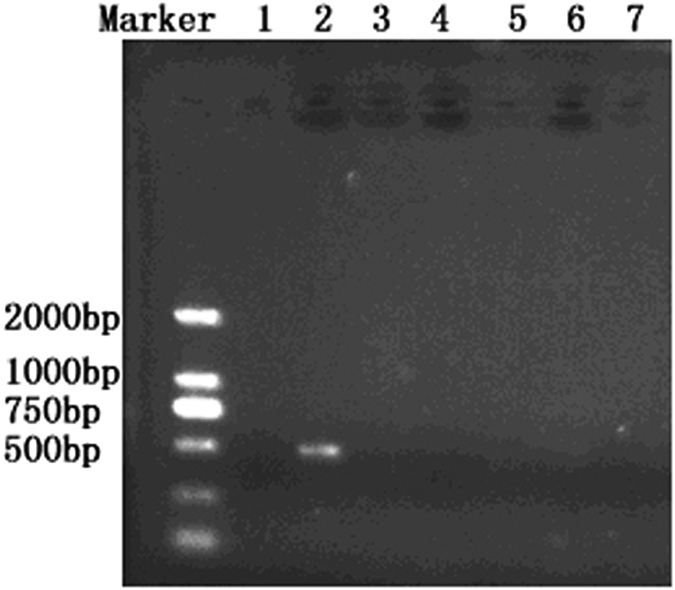
Specificity test of the primers for the target species of *Tilletia.* Lane 1: negative control, Lane 2: *T. horrida* CN1, Lane 3: *T. controversa*, Lane 4: *T. walker*, Lane 5: *T. ehrhartae*, Lane 6: *T. indica*, Lane 7: *T. caries.*

**Figure 3:**
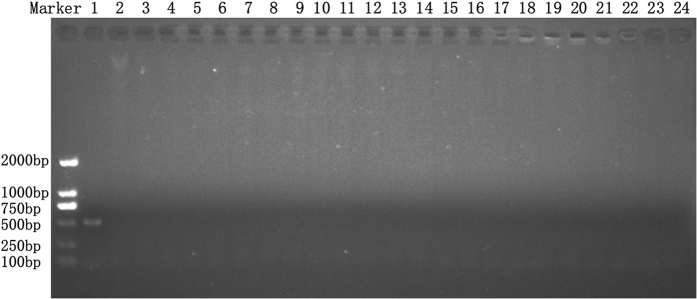
Specificity test of the primers for *Tilletia horrida* and the other fungal genra. Lane 1: Positive control (T*. horrida* CN1), Lane 2: *Pyricularia grisea*, Lane 3: *Ustilaginoidea virens*, Lane 4: *Rhizoctonia solani*, Lane 5: *Fusarium moniliforme*, Lane 6: *Aspergillus flavus*, Lane 7: *Botrytis cinerea*, Lane 8: *Nigrospora sphaerica*, Lane 9: *Alternaria alternata*, Lane 10: *Botryosphaeria dothidea*, Lane 11: *Coniothyrium diplodiella*, Lane 12: *Colletotrichum gloeosporioides*, Lane 13: *Podosphaera leucotricha*, Lane 14: *Glomerella acutata*, Lane 15: *Pestalotiopsis theae*, Lane 16: *Coniella granati*, Lane 17: *Phomopsis fukushii*, Lane 18: *Sclerotinia sclerotiorum*, Lane 19: *Penicillium expansum*, Lane 20: *Monilinia fructicola*, Lane 21: *Gaeumannomyces graminis* Lane 22: *Ascochyta eriobotryae*, Lane 23: *Bipolaris sorokiniana*, Lane 24: Negative control.

The specificity of the *T. horrida* primers was first evaluated using a collection of genomic DNA samples from *T. horrida*, *T. controversa*, *T. walkeri*, *T. ehrhartae*, *T. indica* and *T. caries* as template for PCR assay. The analyzing of amplicons revealed that the designed primers only amplified a 503-bp fragment from the DNA of *T. horrida* but not from the other five tested *Tilletia* species (Fig. 2). Furthermore, when the PCR was performed using genomic DNA of the 22 other fungi from the genera other than *Tilletia* (Table 1), a fragment of the same size could not be amplified (Fig. 3), suggesting that there was no cross species specificity for the PCR assay developed for the detection of *T. horrida*.

### Detection of *T. horrida* in rice seeds

**Figure 4:**
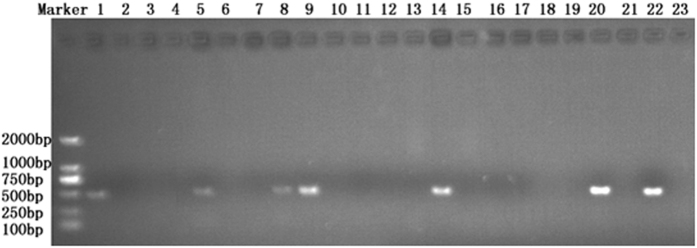
Detection of *Tilletia horrida* in rice seeds. Lane 1–21: Rice seed samples; Lane 22: Positive control (*T. horrida* CN1), Lane 23: Negative Control.

**Figure 5:**
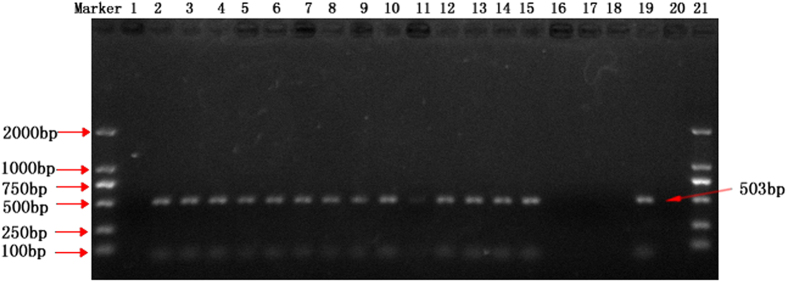
PCR assay for different strains of *Tilletia horrida* and rice seeds. Lane 1 & 20: negative control, Lane 2–12: *Tilletia horrida* strains CN1, HUN-1, AH-1, HEB-1, HIN-1, JS-1, FJ-1, SC-1, YN1, US1, IN1, respectively, Lane 13–15: infected seed samples, Lane 16–18: healthy seed samples, Lane 19: positive control (*T. horrida* CN1).

The optimized PCR assay was performed to evaluate RKS in 21 rice seed samples, out of all these samples, 6 (28.6%) were found to be PCR positive, indicating these were contaminated with the pathogen *T. horrida*, while remaining 15 seed samples were free of the causative pathogen (Fig. 4). These results were also confirmed by the morphological studies of the spores isolated from all the samples under investigation. The specificity of this test was further confirmed by performing PCR using genomic DNA from 11 different strains of *T. horrida* (Table 1) and spores isolated from healthy and infected rice seeds. The diseased status of the seeds was confirmed prior to the experiment from AEEIQB, China. All the 11 strains of *T. horrida*, and infected seeds showed the positive results after agarose gel electrophoresis (Fig. 5). Whereas, no PCR product was obtained in the case of rice seeds with confirmed healthy status.

### Sensitivity of the test

**Figure 6:**
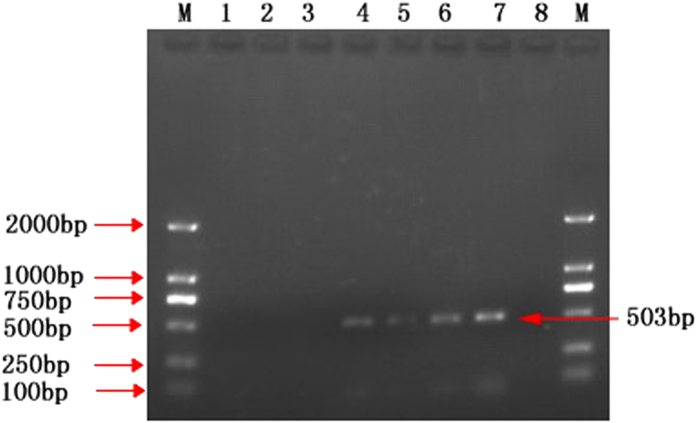
Sensitivity test for the detection of *Tilletia horrida* spores. Lane 1–6: No. of spores (1, 10, 20, 30, 40 & 50, respectively) per 25 μl of reaction mixture; lane 7: positive control (*Tilletia horrida* CN1); lane 8: negative control.

Firstly, the sensitivity of the present method was tested by varying the number of spores isolated from the *T. horrida* contaminated seeds in the PCR reaction mixture. The results depicted that the lower limit for the detection of target pathogen was 30 spores per 25 μl of PCR reaction mixture (Fig. 6). No amplified product was observed in UV illuminator when less than 30 spores were used in the reaction mixture.

**Figure 7:**
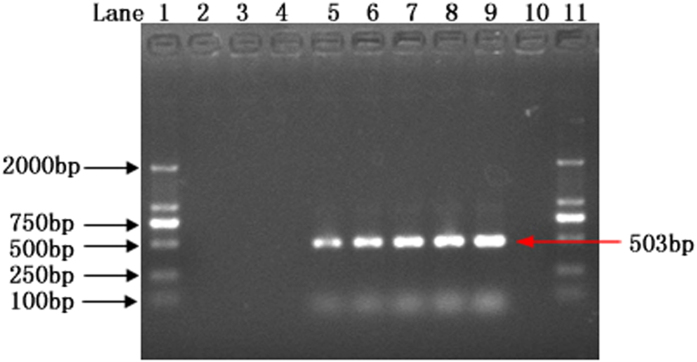
Sensitivity test for the detection of *Tilletia horrida* spores in rice seeds. Lane 1 & 11: DNA ladder; lane 2–8: spore numbers 10, 15, 20, 25, 30, 50 and 100 respectively; lane 9: positive control (*Tilletia horrida* CN1); lane 10: negative control.

Secondly, in order to know the sensitivity of this method for the detection of *T. horrida* in rice seeds, artificially 10, 15, 20, 25, 30, 50 and 100 spores were added into 100 g healthy rice seeds per sample. The results indicated that the lower limit for this method was 25 spores/100 g rice seeds and no PCR product was obtained when less than 20 spores were added in the rice seeds (Fig. 7).

**Figure 8:**
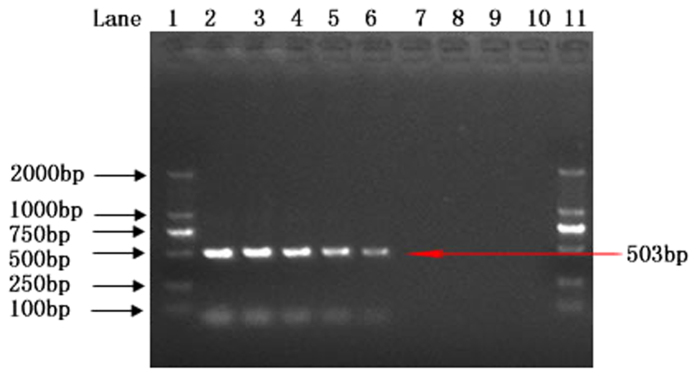
Sensitivity test for the detection of the DNA of *Tilletia horrida.* Lane 1 & 11: DNA ladder; lane 2: positive control; lane 3–9: template DNA concentrations (100 ng, 10 ng, 1 ng, 100 pg, 10 pg, 1 pg and 100 fg, respectively); lane 10: negative control.

Thirdly, to test the sensitivity of this method at the DNA level, the purified DNA (100 ng, 10 ng, 1 ng, 100 pg, 10 pg, 1 pg and 100 fg, respectively) was used as a template for the PCR and the results indicated that this method could detect ≥100 pg genomic DNA of *T. horrida* (Fig. 8).

## Discussion

The emerging epidemic trends throughout the world has placed a greater stress to manage rice kernel smut in the future^9^. Thus, rapid detection and identification of *T. horrida* in rice is crucial for the implementation of surveillance and quarantine regulation in the international rice trade. Detection assays using classical PCR techniques have been developed for numerous plant pathogens, including bacteria, viruses, and fungi^15^. These tests are attractive for several reasons. First, the assays are extremely sensitive and highly specific for the pathogen in question. Second, PCR tests require minimal amounts of sample material, and commercial kits are available for extracting high quality genomic DNA from a wide variety of organisms. Finally, PCR reactions are relatively simple to set up and perform, and results can be obtained quickly, usually within a few hours. In this study, a simple method for the rapid detection and identification of *T. horrida* in rice seeds was developed by the analysis of the ITS region of rDNA from the six *Tilletia* species. The ITS region is long tandem DNA repeat array that is located between ITS1 and ITS2 rRNA genes in the (rDNA) unit in eukaryotes^16^. This region has a high degree of variation even between the closely related species and is widely used for the molecular phylogeny and taxonomic studies. According to a previous study, the ITS region has the highest probability of successful identification for the broadest range of fungi, with the most clearly defined barcode gap between inter- and intraspecific variation among the regions of the ribosomal cistron. ITS will be formally proposed for adoption as the primary fungal barcode marker to the Consortium for the Barcode of Life, with the possibility that supplementary barcodes may be developed for particular narrowly circumscribed taxonomic groups^17^. Other than belonging to the same genus, *T. horrida* is not closely related to the other species included in the study, they possess considerable genomic differences to differentiate them from each other (Fig. 1). Therefore, to distinguish *T. horrida* from other five *Tilletia* species, PCR method was developed by designing a specific pair of primers to amplify a 503 bp fragment of *T. horrida* ITS1 + 5.8 S + ITS2 rDNA region. The designed primers did not amplify any product from the genomic DNA of any of the tested fungi, except *T. horrida* implicating the specificity of the developed PCR protocol. Many other workers have also reported the use of ITS regions to resolve *Tilletia species* comprising closely related *T. horrida*, *T. walker*, and *T. indica*, *T. contraversa*, *T. laevis*, *T. caries*, *T. bromi* and *T. fusca*^14^.

In the last two decades, isozyme analysis was used to distinguish the isolates of *T. indica* from *T. horrida* by using proteins extracted from germinated teliospores. However, considerable experience with the interpretation of complex isozyme polymorphisms associated with *Tilletia* species is required, and this is not considered as a practical approach for the routine identification^18^,^19^,^20^. Several studies have been reported for the identification and differentiation of *T. indica* (a quarantine pathogen causing Karnal bunt of wheat) from *T. walkeri* and *T. horrida*^20^,^21^,^22^ by using conventional PCR with. In 2006, a molecular protocol, using quenched fluorescence resonance energy transfer (FRET) probes, was developed for the detection and differentiation of *T. indica* from *T. walkeri*, *T. horrida* and *T. tritici*^23^. A one-tube fluorescent assay for the quarantine detection and identification of *T. indica* has also been developed^14^. However, all these reports just focused on the detection of *T. indica* and not to distinguish *T. horrida* from the other *Tilletia* species. Moreover, despite of the high sensitivity and accuracy, these methods cannot be commonly used because of the high-priced apparatus and reagent kits. Similarly, other methods such as enzyme-linked immunosorbent assay (ELISA)^24^, sequence-characterized amplified region (SCAR)^25^ inter simple sequence repeat (ISSR) marker^26^ have also been developed for the diagnosis of *T. caries* and *T. controversa*, respectively. However, these methods are either expensive or time-consuming.

Compared to these methods, the method for detection and differentiation of *T. horrida* from other *Tilletia* species in our study possesses numerous advantages. It depends on common techniques of molecular biology and needs merely basic PCR reagents, electrophoresis apparatus and thermocycler. This equipment is relatively cheap, inexpensive to maintain, and simple to manipulate. In our method we report the direct use of teliospores for the diagnosis of *T. horrida* without the culturing and genomic DNA isolation. Thus eliminating the lengthy culturing and difficult morphological identification procedures^27^,^28^. Furthermore, sensitivity test results showed that the present method can be employed using only ~30 spores in the reaction mixture. The traditional morphological identification methods usually require considerable expertise and a significant of ~50 spores for statistical determination^14^,^29^. The potential benefits of this technology can be especially recognized in the regulatory field, where both the timeliness and accuracy of identifications are crucial^20^,^30^.

In our study, the time required for this detection is less than three hours, therefore, this developed technique can be used to screen out a large number of samples in a short time. Hence, it will be extremely useful to resolve the disputes regarding contamination of rice with smut teliospores. In this study, we have also confirmed the identity of the pathogen of the kernel smut of rice using the specifically designed primers.

This information would be useful in establishing quarantine areas and preventing the contamination of clean rice shipments with teliospores from infested grains.

## Methods

### Fungal strains

All the fungal strains (Table 1) used in the present study were provided by the Anhui Entry-Exit Inspection and Quarantine Bureau (AEEIQB), China. The fungal cultures were maintained on potato dextrose agar (PDA) medium and stored at 4 °C.

### Isolation of *T. horrida* spores

The rice seed samples used for the detection and isolation of *T. horrida* spores were also supplied by the courtesy of AEEIQB, China that were collected from the different geographic locations (Table 1). The rice seeds were surface-sterilized in 0.1% sodium hypochlorite for 5 min, rinsed in sterile distilled water for 30 sec twice, striped and the teliospores scraped into a clear tube standby. The isolated spores were identified and confirmed by AEEIQB, China using Chinese National Standard^31^ for the identification of *T. horrida*. The spore counting was performed using hemocytometer.

### Extraction of genomic DNA

Genomic DNA of *T. horrida* was extracted directly from the isolated spores using the Fungal DNA Kit (Omega Bio-Tek) according to the manufacturer’s protocol. Whereas, the genomic DNAs of the other tested pathogens (Table 1) were extracted from 50 mg mycelia using the same kit. The spores and mycelia were treated with a minibeadbeater (607EUR, Biospec, USA) before isolation of the DNA. The DNA concentration for each sample was determined by Nanodrop (NanoVue Plus, GE Healthcare Life Sciences).

### Primer designing and PCR amplification

To design the primers, Internal Transcribed Spacer (ITS) sequence of ribosomal DNA of *T. horrida,* Accession No. DQ827699.1^32^ was retrieved from the NCBI database. The sequence was compared and analyzed with the other *Tilletia* species sequences from the NCBI database using online tools BLASTN^33^ and Clustal Omega^34^. The forward primer (HF-F): 5′-GAGAGTCAACTTATGTTCA-3′ and reverse primer (HF-R): 5′-GATGAAAGTTACTCTCAT-3′ were designed by using the Bioedit software (v7.0.5) to amplify 503 bp fragment of 5.8 S ribosomal DNA (rDNA) of *T. horrida*. The PCR conditions were optimized using *T. horrida* CN1 as template DNA. The DNA amplification was carried out in DNA Engine System PT-200 (Bio-Rad) using 25 μl reaction mixture containing 1 U ExTaq polymerase, 2.5 μl of 10× ExTaq buffer, 2 μl Mg^2+^ (25 mM), 200 μm of dNTPs, 1 μm of each primer and DNA template (50 ng). The thermal cycler was programed for one cycle of initial denaturation of 5 min at 94 °C; followed by 30 cycles of 1 min at 94 °C, 30 sec at 50 °C and 30 sec at 72 °C with a final extension for 5 min at 72 °C; and a holding temperature of 4 °C. A negative control, replacing the DNA template with sterile distilled water and DNA ladder DL2000 were also run in parallel. The PCR products were electrophoresed using 1% agarose gel in 1xTAE buffer for 45 min at 90 V to analyze the results.

### Specificity of the test

To determine the specificity of the PCR and primers to *T. horria*, the PCR was carried out with different species of *Tilletia* and other common contaminant fungal strains (Table 1) using the above mentioned optimized PCR conditions.

### Detection of *T. horrida* in rice seeds

A total of 21 rice seed samples, provided by AEEIQB, China, were used for the detection of *T. horrida*. For each sample, 10 g of the seeds were washed with sterile distilled water for 30 sec twice and saturated in sterile distilled water for 5 min. The saturated water was centrifuged at 12000 rpm for 10 min, then discarded the supernatant and re-suspended the pellet in 50 μl water. 10 μl of this spore suspension was directly used as template DNA for the PCR.

### Sensitivity of the test

Firstly, the sensitivity test for the detection *T. horrida* contaminated seeds was performed by varying the number of spores per 25 μl of PCR reaction mixture. PCR was performed using individual reaction mixtures containing 1, 10, 20, 30, 40 & 50 spores of *T. horrida*, respectively, followed by agarose gel electrophoresis analysis.

Secondly, artificially 10, 15, 20, 25, 30, 50 and 100 spores were added into the individual 100 g healthy rice seeds samples and then, the spores were collected by seed washing and centrifugation. The PCR was performed using the individual spore suspensions.

Thirdly, in order to know the sensitivity of this PCR method for DNA limit, the purified DNA (100 ng, 10 ng, 1 ng, 100 pg, 10 pg, 1 pg and 100 fg, respectively) was used as a template in the indicidual PCR assays followed by the same cycling conditions as described above.

## Additional Information

**How to cite this article**: Chen, Y. *et al.* Simple and rapid detection of *Tilletia horrida* causing rice kernel smut in rice seeds. *Sci. Rep.* **6**, 33258; doi: 10.1038/srep33258 (2016).

## Figures and Tables

**Table 1:** List of fungal species, their hosts and geographical origin.

Taxon	Specimen Number	Host	Geographic origin
*T. horrida*	CN1	Rice	Jiangsu, China
*T. horrida*	HUN-1	Rice	HuNan, China
*T. horrida*	AH-1	Rice	Anhui, China
*T. horrida*	HEB-1	Rice	Hebei, China
*T. horrida*	HIN-1	Rice	Hainan, China
*T. horrida*	JS-1	Rice	Jiangsu, China
*T. horrida*	FJ-1	Rice	Fujian, China
*T. horrida*	SC-1	Rice	Sichuan, China
*T. horrida*	YN1	Rice	Yunnan, China
*T. horrida*	US1	Rice	USA
*T. horrida*	IN1	Rice	India
*T. controversa*	VC01	Barley	USA
*T. caries*	DL01	Wheat	Liaoning, China
*T. walkeri*	CA21	Ryegrass	USA
*T. indica*	FI01	Wheat	India
*T. ehrhartae*	TEA01	Grass	Australia
*Alternaria alternata*	DSHB1	Pear	Anhui, China
*Ascochyta eriobotryae*	HS-1	Loquat	Anhui, China
*Aspergillus flavus*	HUB01	Rice	Anhui, China
*Bipolaris sorokiniana*	WGF-1	Wheat	Anhui, China
*Botryosphaeria dothidea*	PKY-1	Grape	Anhui, China
*Botrytis cinerea*	FQ13	Tomato	Anhui, China
*Colletotrichum gloeosporioides*	LTJ-1	Pear	Anhui, China
*Coniella granati*	SGF-1	Pomegranate	Anhui, China
*Coniothyrium diplodiella*	PBF-1	Grape	Anhui, China
*Fusarium moniliforme*	FY04S	Wheat	Anhui, China
*Gaeumannomyces graminis*	QS-4	Wheat	Anhui, China
*Glomerella acutata*	LJTJ-2	Pepper	Anhui, China
*Monilinia fructicola*	THF-1	Peach	Anhui, China
*Nigrospora sphaerica*	MHB-1	Kiwifruit	Anhui, China
*Penicillium expansum*	TQM-3	Peach	Anhui, China
*Pestalotiopsis theae*	PHB-1	Loquat	Anhui, China
*Phomopsis fukushii*	LGK-1	Pear	Anhui, China
*Podosphaera leucotricha*	CMBF-1	Strawberry	Anhui, China
*Pyricularia grisea*	DW-1	Rice	Anhui, China
*Rhizoctonia solani*	DWK-7	Rice	Anhui, China
*Sclerotinia sclerotiorum*	LHF-1	Pear	Anhui, China
*Ustilaginoidea virens*	DQ-12	Rice	Anhui, China

## References

[b1] Sharif M. K., Butt M. S., Anjum F. M. & Khan S. H. Rice bran: a novel functional ingredient. Crit. Rev. Food Sci. Nutr. 54, 807–816 (2014). 24345050 10.1080/10408398.2011.608586

[b2] Wu J. G., Shi C. & Zhang X. Estimating the amino acid composition in milled rice by near-infrared reflectance spectroscopy. F. Crop. Res. 75, 1–7 (2002).

[b3] Goff S. A. *et al.* A draft sequence of the rice genome (*Oryza sativa* L. ssp. *japonica*). Science 296, 92–100 (2002). 11935018 10.1126/science.1068275

[b4] Webster R. K. & Gunnell P. S. Compendium of rice diseases. APS Press 62 (1992).

[b5] Carris L. M., Castlebury L. A. & Goates B. J. Nonsystemic Bunt Fungi - *Tilletia indica* and *T. horrida*: A Review of History, Systematics, and Biology. Annu. Rev. Phytopathol. 44, 113–133 (2006). 16480336 10.1146/annurev.phyto.44.070505.143402

[b6] Wang N. *et al.* Draft Genome Sequence of the Rice Kernel Smut *Tilletia horrida* Strain QB-1. Genome Announc. 3, (2015). 10.1128/genomeA.00621-15 PMC4481280 26112782

[b7] Anders M. M., Brooks S., Yeater K. M., Watkins K. B. & Mccarty D. Reducing False Smut (Ustilaginoidea virens) and Kernel Smut (Neovossia horrida) Disease Severity Through Crop Management Practices. AAES Res. Ser. 571 B.R. Wells Rice Res. Stud. at http://arkansasagnews.uark.edu/4300.htm (2008).

[b8] Tsuda M., Sasahara M., Ohara T. & Kato S. Optimal application timing of simeconazole granules for control of rice kernel smut and false smut. J. Gen. Plant Pathol. 72, 301–304 (2006).

[b9] Brooks S. A., Anders M. M. & Yeater K. M. Effect of Cultural Management Practices on the Severity of False Smut and Kernel Smut of Rice. Plant Dis. 93, 1202–1208 (2009). 10.1094/PDIS-93-11-1202 30754580

[b10] Leung H. *et al.* Using Genetic Diversity to Achieve Sustainable Rice Disease Management. Plant Dis. 87, 1156–1169 (2003). 10.1094/PDIS.2003.87.10.1156 30812716

[b11] Slaton N. A. *et al.* Grain Yield and Kernel Smut of Rice as Affected by Preflood and Midseason Nitrogen Fertilization in Arkansas. Agron. J. 96, 91–99 (2004).

[b12] Whitney N. G. Effect of fungicide applications on kernel smut of rice [*Neovossia horrida*]. Plant Dis. Report. 61, 379–381 (1977).

[b13] Chen Y. *et al.* Simple and rapid detection of rice false smut pathogen *Ustilaginoidea virens* in rice seeds. Phytoparasitica 42, 371–375 (2014).

[b14] Tan M.-K. *et al.* A one-tube fluorescent assay for the quarantine detection and identification of *Tilletia indica* and other grass bunts in wheat. Australas. Plant Pathol. 38, 101 (2009).

[b15] Henson J. M. & French R. The polymerase chain reaction and plant disease diagnosis. Annu. Rev. Phytopathol. 31, 81–109 (1993). 18643762 10.1146/annurev.py.31.090193.000501

[b16] Rogers S. O. & Bendich A. J. Ribosomal RNA genes in plants: variability in copy number and in the intergenic spacer. Plant Mol. Biol. 9, 509–520 (1987). 24277137 10.1007/BF00015882

[b17] Schoch C. L. *et al.* Nuclear ribosomal internal transcribed spacer (ITS) region as a universal DNA barcode marker for Fungi. Proc. Natl. Acad. Sci. USA 109, 6241–6246 (2012). 22454494 10.1073/pnas.1117018109 PMC3341068

[b18] Bonde M. R., Peterson G. L. & Royer M. H. Inheritance of isoenzymes in the smut pathogen *Tilletia indica*. Phytopathology 8, 1276–1279 (1988).

[b19] Bonde M. R., Peterson G. L. & Matsunoto T. T. The use of isoenzyme to identify teliospores of *Tilletia indica*. Phytopathology 79, 596–599 (1989).

[b20] Smith O., Peterson G., Beck R., Schaad N. & Bonde M. Development of a PCR-based method for identification of *Tilletia indica*, causal agent of Karnal bunt of wheat. Phytopathology 86, 115–122 (1996).

[b21] Frederick R. D. *et al.* Identification and Differentiation of *Tilletia indica* and *T. walkeri* Using the Polymerase Chain Reaction. Phytopathology 90, 951–960 (2000). 18944518 10.1094/PHYTO.2000.90.9.951

[b22] Levy L., Castlebury L. A., Carris L. M., Meyer R. J. & Pimentel G. Internal Transcribed Spacer Sequence-Based Phylogeny and Polymerase Chain Reaction-Restriction Fragment Length Polymorphism Differentiation of *Tilletia walkeri* and *T. indica*. Phytopathology 91, 935–940 (2001). 18944119 10.1094/PHYTO.2001.91.10.935

[b23] Tan M. K. & Murray G. M. A molecular protocol using quenched FRET probes for the quarantine surveillance of *Tilletia indica*, the causal agent of Karnal bunt of wheat. Mycol. Res. 110, 203–210 (2006). 16388942 10.1016/j.mycres.2005.08.006

[b24] Eibel P., Wolf G. A. & Koch E. Detection of *Tilletia caries*, causal agent of common bunt of wheat, by ELISA and PCR. J. Phytopathol. 153, 297–306 (2005).

[b25] Gao L., Chen W. & Liu T. An ISSR-based Approach for the Molecular Detection and Diagnosis of Dwarf Bunt of Wheat, Caused by *Tilletia controversa* Kühn. J. Phytopathol. 159, 155–158 (2011).

[b26] Liu J. H., Gao L., Liu T. G. & Chen W. Q. Development of a sequence-characterized amplified region marker for diagnosis of dwarf bunt of wheat and detection of *Tilletia controversa* Kühn. Lett. Appl. Microbiol. 49, 235–240 (2009). 19486286 10.1111/j.1472-765X.2009.02645.x

[b27] Hamelin R. C., Ouellette G. B. & Bernier L. Identification of Gremmeniella abietina races with random amplified polymorphic DNA markers. Appl. Environ. Microbiol. 59, 1752–1755 (1993). 16348951 10.1128/aem.59.6.1752-1755.1993 PMC182156

[b28] Zhang A. W., Hartman G. L., Curio-Penny B., Pedersen W. L. & Becker K. B. Molecular Detection of *Diaporthe phaseolorum* and *Phomopsis longicolla* from Soybean Seeds. Phytopathol. 89, 796–804 (1999). 10.1094/PHYTO.1999.89.9.796 18944708

[b29] Inman A., Hughes K. & Bowyer R. EU recommended protocol for the diagnosis of a quarantine pathogen, Tilletia indica. (Central Sciences Laboratory, 2003).

[b30] Goodwin P. H. & Annis S. L. Rapid identification of genetic variation and pathotype of Leptosphaeria maculans by random amplified polymorphic DNA assay. Appl. Environ. Microbiol. 57, 2482–2486 (1991). 1768121 10.1128/aem.57.9.2482-2486.1991 PMC183606

[b31] Wu P.-S., Luo J.-F., Du H.-Z. & Yan J. National Standard of China (GB/T 28079-2011) — Detection and identification of *Tilletia horrida* Tak. (ICS 65. 020. 01, B 16).

[b32] Zhou Y. *et al.* PCR-based specific detection of *Ustilaginoidea virens* and *Ephelis japonica*. J. Phytopathol. 151, 513–518 (2003).

[b33] Altschul S. F. *et al.* Gapped BLAST and PSI-BLAST: A new generation of protein database search programs. Nucleic Acids Res. 25, 3389–3402 (1997). 9254694 10.1093/nar/25.17.3389 PMC146917

[b34] McWilliam H. *et al.* Analysis Tool Web Services from the EMBL-EBI. Nucleic Acids Res. 41 (2013). 10.1093/nar/gkt376 PMC3692137 23671338

